# TRF1 Depletion Reveals Mutual Regulation Between Telomeres, Kinetochores, and Inner Centromeres in Mouse Oocytes

**DOI:** 10.3389/fcell.2021.749116

**Published:** 2021-09-17

**Authors:** Hyuk-Joon Jeon, Jeong Su Oh

**Affiliations:** Department of Integrative Biotechnology, College of Biotechnology and Bioengineering, Sungkyunkwan University, Suwon, South Korea

**Keywords:** telomere, centromere, kinetochore, TRF1, oocyte

## Abstract

In eukaryotic chromosomes, the centromere and telomere are two specialized structures that are essential for chromosome stability and segregation. Although centromeres and telomeres often are located in close proximity to form telocentric chromosomes in mice, it remained unclear whether these two structures influence each other. Here we show that TRF1 is required for inner centromere and kinetochore assembly in addition to its role in telomere protection in mouse oocytes. TRF1 depletion caused premature chromosome segregation by abrogating the spindle assembly checkpoint (SAC) and impairing kinetochore-microtubule (kMT) attachment, which increased the incidence of aneuploidy. Notably, TRF1 depletion disturbed the localization of Survivin and Ndc80/Hec1 at inner centromeres and kinetochores, respectively. Moreover, SMC3 and SMC4 levels significantly decreased after TRF1 depletion, suggesting that TRF1 is involved in chromosome cohesion and condensation. Importantly, inhibition of inner centromere or kinetochore function led to a significant decrease in TRF1 level and telomere shortening. Therefore, our results suggest that telomere integrity is required to preserve inner centromere and kinetochore architectures, and vice versa, suggesting mutual regulation between telomeres and centromeres.

## Introduction

Accurate chromosome segregation is essential for genome integrity, and successful completion of this process relies on dramatic changes in chromosome organization during the cell cycle. Unlike mitosis, meiosis consists of two rounds of chromosome segregation following a single round of DNA replication. During meiosis I, chromatin condenses into compact structures, and sister chromatids are kept together by the cohesin complex, generating sister chromatid cohesion around the centromeres and along the chromosome arms (Wood et al., 2010). As a consequence of sister chromatid cohesion on the chromosome arms and reciprocal recombination between homologous chromosomes, homologous chromosomes become linked physically. These physically connected partner chromosomes form a bivalent species and attach to the spindle microtubules through their kinetochores and centromeres. Unattached or improperly attached kinetochores activate the spindle assembly checkpoint (SAC) to delay anaphase onset (Foley and Kapoor, 2013). Furthermore, centromeric Aurora B/C kinases in complex with INCENP, Survivin, and Borealin regulate kinetochore-microtubule (kMT) attachment through phosphorylation of multiple kinetochore proteins (DeLuca et al., 2006; Ruchaud et al., 2007; Welburn et al., 2010). Once all bivalents are attached successfully to the bipolar spindle microtubules with proper tension and aligned well in the middle of the metaphase plate, they are segregated into univalents. Therefore, the dynamics of chromosome organization during the cell cycle, including condensation, cohesion, and centromere/kinetochore assembly, are crucial to ensure stable kMT attachment and reliable chromosome segregation.

Although the number and size of chromosomes can vary by species, all linear eukaryotic chromosomes contain centromeres and telomeres. These specialized structures are essential for chromosome stability and segregation. The centromere is the specialized DNA region containing histone H3 variant CENP-A (Schalch and Steiner, 2017). Although CENP-A forms specialized chromatin that is essential for kinetochore assembly, the exact composition and nature of CENP-A-containing nucleosomes *in vivo* remain elusive (Henikoff and Furuyama, 2010; Black and Cleveland, 2011). The constitutive centromere-associated network (CCAN) loads onto centromeric chromatin and is important for outer kinetochore assembly (Perpelescu and Fukagawa, 2011). The outer kinetochore consists of the conserved KMN network (Knl1, Mis12, Ndc80/Hec1 complexes) that serves as a core microtubule-binding module (Cheeseman et al., 2006). The centromere and kinetochore also provide structural platforms for many regulatory proteins, including SAC, motor proteins, microtubule-associated proteins (MAPs), and protein kinases and phosphatases. Inner centromeric localization of Aurora B/C kinases, which is dependent on histone H3 phosphorylation at Thr-3 (H3T3ph), is essential to maintain the connection of sister chromatids by mediating assembly of the cohesin complex for proper chromosome segregation (Dai et al., 2006; Tanaka et al., 2013). In addition, Mps1 kinase, a key component of the SAC, regulates chromosome alignment and segregation at the kinetochores (Abrieu et al., 2001). Therefore, a loss in centromere identity results in aberrant chromosome segregation caused by a misalignment of chromosomes to spindle microtubules (Tanaka et al., 2013; McKinley and Cheeseman, 2016). On the other hand, the telomere is a region of repetitive DNA sequences associated with specialized proteins at the ends of linear chromosomes (O’Sullivan and Karlseder, 2010). One of these telomeric protein complexes is known as shelterin, composed of six individual proteins identified as TRF1, TRF2, TIN2, TPP1, RAP1, and POT1 (de Lange, 2005). Telomeres protect chromosome ends from degradation and fusion, securing genome stability and integrity (O’Sullivan and Karlseder, 2010). Telomere loss or dysfunction can cause chromosomal instability, leading to malignant cancer and poor clinical outcomes (Artandi et al., 2000; Gisselsson et al., 2001).

The typical karyotype of mouse chromosomes is telocentric, having no obvious short arm at the cytogenetic level (Pialek et al., 2005). Therefore, the telomere is located in close proximity to the centromere in mouse chromosomes. However, it is unclear whether these two chromosomal structures impact each other. In the present study, we showed that disruption of telomere structure by TRF1 depletion impaired inner centromere and kinetochore function, and vice versa, suggesting mutual regulation between telomeres and centromeres to maintain chromosome integrity.

## Materials and Methods

### Oocyte Collection and Culture

Female three-week-old ICR mice (Koatech, South Korea) were used in all experiments. Experiments were approved by the Institutional Animal Care and Use Committees of Sungkyunkwan University (approval ID: SKKUIACUC2020-05-01-1). Cumulus-enclosed oocytes were isolated from the ovaries of female mice primed with 5 IU pregnant mare serum gonadotropin (PMSG) at 46–48 h before sample collection. After removing cumulus cells by pipetting, oocytes were cultured in M2 medium supplemented with 200 μM 3-isobutyl-1-methylxanthine (IBMX) to prevent meiotic resumption. Regarding *in vitro* maturation, oocytes were cultured in IBMX-free M2 medium under mineral oil at 37°C in a 5% CO_2_ incubator. Oocytes at metaphase I (MI) were incubated in ice-cold M2 medium for 10 min for analysis of kMT attachment. After cold treatment, oocytes were fixed and subjected to immunostaining.

Regarding chemical treatment, MI oocytes were treated with 400 nM nocodazole, 2 μM AZ3146 (Selleck Chemicals), or 10 μM ZM447439 (Selleck Chemicals). All chemicals and culture media were purchased from Sigma-Aldrich unless stated otherwise.

### Microinjection

Double-stranded RNAs (dsRNAs) were synthesized using the MEGAscript T7 Transcription Kit (Ambion). The templates used for dsRNA synthesis were PCR products amplified using the following primers: EGFP, ATTAATACGACTAACTATAGGGAGAATGGTGAGCAAGGG CGAG and ATTAATACGACTCACTATAGGGAGAGCTCGTCC ATGCCGAGAG; TRF1, ATTAATACGACTCACTATAGGGAGA CCTTGTGGCTGAGGTGGAGGC and ATTAATACGACTCACTATAGGGAGACTCGCTTTCTCATTT TCCACTACTTTTGTTGCTG. After purification, approximately 5–10 pl of dsRNA was microinjected into the cytoplasm of oocytes using a FemtoJet microinjector (Eppendorf, Germany) with a Leica inverted microscope (DMIRB) equipped with a micromanipulator (Narishige, Japan). After injection, oocytes were cultured for 24 h in a medium containing IBMX. The oocytes were transferred to fresh medium and cultured under mineral oil at 37°C in an atmosphere of 5% CO_2_.

For Hec1 Trim-away, oocytes were injected with mRNA encoding Trim21-mCherry. After 1 h of culture in IBMX-containing M2 medium, oocytes were cultured in IBMX-free medium for 5 h and microinjected with Hec1 or IgG antibodies. Trim21-mCherry mRNA was prepared as described previously (Clift et al., 2017).

### Immunostaining

Oocytes were fixed in 4% paraformaldehyde for 20 min and permeabilized in phosphate-buffered saline (PBS) with 0.25% Triton X-100 for 30 min. After permeabilization, oocytes were blocked in 3% BSA in PBS for 1 h at room temperature. Oocytes were incubated overnight at 4°C with primary antibodies and then at room temperature for 2 h with secondary antibodies. Chromosomes were counterstained with DAPI. Oocytes were examined under a confocal laser scanning microscope (LSM 700; Zeiss, Germany) equipped with a C-Apochromat 40x/1.2 water immersion objective. For each experiment, the intensity settings were not changed between sample groups. ZEN LSM software (Zeiss, Germany) was used to measure and analyze fluorescence intensity. The fluorescence intensity was normalized to the mean intensity of DAPI signal unless stated otherwise, and data are presented as normalized fluorescence compared to control. Data were obtained from at least three independent experiments unless otherwise specified, and each experimental group included at least 15 oocytes.

### Antibodies

Primary antibodies used for immunostaining were anti-TRF1 (Abcam, ab192629, 1:100), anti-BubR1 (Abcam, ab28193, 1:100), anti-Zw10 (Abcam, ab21582, 1:100), anti-acetylated-α-tubulin (Sigma Aldrich, T7451, 1:500; Abcam, ab179484, 1:500), anti-centromere (Antibodies Incorporated, 15-234, 1:100), anti-CENP-A (Cell Signaling, #2048, 1:100), anti-Survivin (Cell Signaling, #2808, 1:100), anti-Hec1 (Santa Cruz Biotechnology, sc-515550, 1:100), anti-H3T3ph (Upstate, 07-424, 1:100), anti-SMC3 (Abcam, ab128919, 1:100), anti-SMC4 (Novus Biologicals, NBP1-86635, 1:100), and anti-TRF2 (Abcam, ab13579, 1:100). Secondary antibodies were Alexa Fluor 488-conjugated anti-mouse (Jackson ImmunoResearch, 115-545-144 1:500), Alexa Fluor 594-conjugated anti-mouse (Jackson ImmunoResearch, 111-585-146, 1:500), Alexa Fluor 488-conjugated anti-rabbit (Jackson ImmunoResearch, 115-545-144 1:500), Alexa Fluor 594-conjugated anti-rabbit (Jackson ImmunoResearch, 111-585-144, 1:500), and Alexa Fluor 488-conjugated anti-sheep antibodies (Abcam, ab150177, 1:500).

### Chromosome Spreading

Oocytes were exposed to acidic Tyrode’s solution (pH 2.5) for 1 min to remove the zona pellucida. After a brief recovery in fresh medium, oocytes were fixed in 1% paraformaldehyde in distilled water (pH 9.2) containing 0.15% Triton X-100 and 3 mM dithiothreitol. The slides were dried slowly in a humid chamber for several hours and then blocked with 1% BSA in PBS for 1 h at room temperature. Oocytes were incubated with a primary antibody overnight at 4°C and then with a secondary antibody for 2 h at room temperature. DNA was stained with DAPI, and the slides were mounted for observation by confocal microscopy.

### Telomere Quantitative-Fluorescence *in situ* Hybridization

Oocytes were exposed to acidic Tyrode’s solution (pH 2.5) for 1 min to remove the zona pellucida. After a brief recovery in fresh medium, oocytes were treated with hypotonic solution (1% sodium citrate) for 15 min, followed by fixation in methanol: acetic acid (3:1) for 30 min and spreading on a glass slide. Telomeres were denatured at 80°C for 5 min and hybridized with Cy3-labeled (CCCTAA)3 peptide nucleic acid (PNA) probes at 200 nM (Panagene, South Korea). After counterstaining DNA with DAPI, telomeres were examined under a confocal laser-scanning microscope (LSM 700) equipped with a C-Apochromat 63x/1.2 water immersion objective. For each experiment, the intensity settings were not changed between sample groups. The fluorescence intensity of telomeres was normalized to the mean intensity of DAPI signal. Data were obtained from at least three independent experiments unless otherwise specified, and each experimental group included at least 15 chromosome spreads.

### Telomere Quantitative-Polymerase Chain Reaction

Telomere quantitative-polymerase chain reaction (Q-PCR) was performed as described previously (Wang et al., 2013). Briefly, MII oocytes were exposed to acidic Tyrode’s solution (pH 2.5) for 1 min to remove the zona pellucida. After a brief recovery in fresh medium, oocytes were separated from polar bodies by mechanical pipetting. After washing in PBS, the oocytes were isolated and lysed in a PCR tube without physical purification. Polymerase chain reaction was performed using telomere primers and reference control gene primers (mouse 36B4 single-copy gene), and the previously described PCR settings were used (Wang et al., 2013). Relative telomere length was determined by calculating the telomere to 36B4 single-copy gene ratio (T/S ratio) from at least five replicates for each group.

### Statistical Analysis

Statistical analysis was performed with GraphPad Prism 5.0 (GraphPad Software Inc.). The data are presented as the mean ± SEM of at least three independent experiments unless otherwise stated. Differences between two groups were analyzed by Student’s *t*-test, and comparisons between more than two groups were analyzed by one-way ANOVA with Tukey’s *post hoc* test. *P* < 0.05 was considered statistically significant.

## Results

### TRF1 Depletion Causes Precocious Polar Body Extrusion by Abrogating Spindle Assembly Checkpoint

**FIGURE 1:**
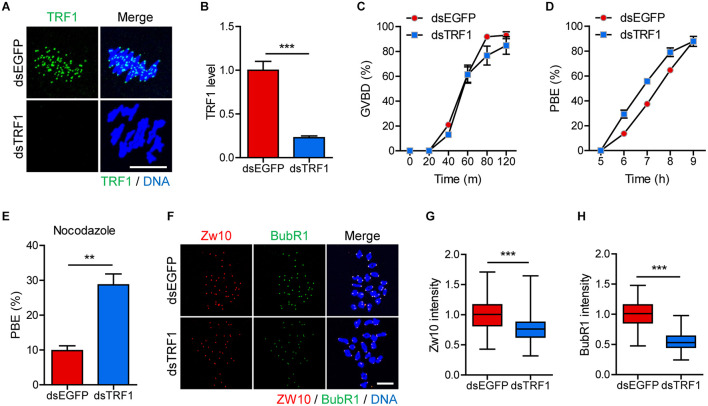
TRF1 depletion accelerates polar body extrusion by abrogating the SAC. After microinjection of TRF1 dsRNA (dsTRF1) or control EGFP dsRNA (dsEGFP), the oocytes were cultured in M2 medium containing IBMX for 24 h and then transferred to IBMX-free M2 medium for up to 16 h. **(A,B)** Oocytes at MI stage (8 h after IBMX release) were fixed and stained with anti-TRF1 antibody. DNA was counterstained with DAPI (scale bar, 10 μm). TRF1 intensity was quantified and shown as mean ± SEM from at least four independent experiments with representative images. **(C,D)** Rates of GVBD and polar body extrusion (PBE) were scored. **(E)** Oocytes at the MI stage (8 h after IBMX release) were cultured in medium containing 20 μg/ml nocodazole and were scored 6 h later for PBE. **(F–H)** Chromosome spreads were prepared from oocytes at the MI stage and were stained with anti-Zw10 and anti-BubR1 antibodies. DNA was counterstained with DAPI (scale bar, 20 μm). The intensity of Zw10 and BubR1 was quantified from three independent experiments and shown with representative images. **p < 0.01, ***p < 0.001.

We initially examined the localization of TRF1 during meiotic maturation in mouse oocytes. As described previously (Munoz et al., 2009), TRF1 signals appeared in the proximity of kinetochores at centromeric ends (Supplementary Figure 1). To investigate the effect of telomeres on centromere and kinetochore architectures, we depleted TRF1 by injecting double-stranded RNA (dsRNA) targeting TRF1 (dsTRF1). Control oocytes were injected with EGFP dsRNA (dsEGFP). Immunostaining of TRF1 revealed that the expression of TRF1 was significantly reduced following dsTRF1 injection (1.0 ± 0.10, *n* = 40 vs. 0.22 ± 0.02, *n* = 40, *p* < 0.0001) (Figures 1A,B). Interestingly, TRF1 depletion resulted in a partial reduction in TRF2 at chromosome ends and induced telomere shortening (TRF2 level: 1.0 ± 0.02, *n* = 285 vs. 0.69 ± 0.02, *n* = 236, *p* < 0.0001; relative telomere length: 1.05 ± 0.05, *n* = 3 vs. 0.4 ± 0.1, *n* = 3, *p* < 0.05) (Supplementary Figure 2). Therefore, these results suggest that TRF1 is required to maintain telomere length and structural integrity in mouse oocytes. We next investigated the impact of TRF1 depletion on meiotic maturation in oocytes. TRF1-depleted oocytes underwent GVBD with kinetics comparable to those of control oocytes (at 120 min: 92.97 ± 2.76, *n* = 60 vs. 84.72 ± 7.01, *n* = 60, *p* > 0.05) (Figure 1C). However, the timing of polar body extrusion (PBE) was accelerated after TRF1 depletion, despite similar PBE rates (at 7 h: 37.5 ± 1.44, *n* = 60 vs. 55.66 ± 1.2, *n* = 60, *p* < 0.001; at 9 h: 88.05 ± 1.0, *n* = 60 vs. 87.81 ± 3.97, *n* = 60, *p* > 0.05) (Figure 1D). This result implies that SAC activity is compromised after TRF1 depletion. Indeed, TRF1-depleted oocytes were able to override the MI arrest induced by nocodazole (9.82 ± 1.38, *n* = 50 vs. 28.69 ± 3.13, *n* = 50, *p* < 0.01) (Figure 1E). To further confirm defective SAC activity in TRF1-depleted oocytes, we examined BubR1 and Zw10 levels. Oocyte chromosome spread at 8 h following IBMX release showed that both BubR1 and Zw10 levels at the kinetochores significantly decreased after TRF1 depletion (for Zw10: 1.00 ± 0.02, *n* = 118 vs. 0.77 ± 0.02, *n* = 108, *p* < 0.0001; for BubR1: 1.00 ± 0.02, *n* = 118 vs. 0.55 ± 0.01, *n* = 108, *p* < 0.0001) (Figures 1F–H). Therefore, TRF1 likely regulates SAC activity and is required to prevent premature chromosome segregation in mouse oocytes.

### TRF1 Depletion Compromises kMT Attachment and Increases the Incidence of Aneuploidy

**FIGURE 2:**
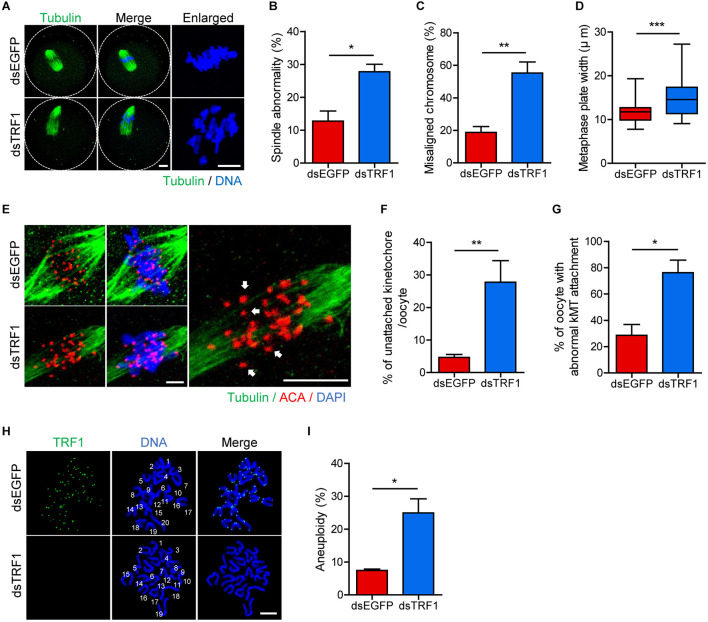
TRF1 depletion impairs kMT attachment and increases the rate of aneuploidy. Oocytes injected with dsEGFP or dsTRF1 were cultured in medium containing IBMX for 24 h and then transferred to IBMX-free medium for 8 h. **(A–D)** Oocytes at the MI stage were fixed and stained with anti-α-tubulin antibody and DAPI (scale bar, 10 μm). Spindle abnormality, chromosome misalignment, and metaphase plate width were quantified and are shown in representative images. Data are presented as mean ± SEM from three independent experiments. **(E–G)** After cold treatment, oocytes were fixed and stained with anti-centromere antibody (ACA), anti-α-tubulin antibody, and DAPI to visualize kinetochore, spindle, and DNA, respectively. The percentages of unattached kinetochores per oocyte and oocytes with abnormal kMT attachment were quantified, also shown in representative images (scale bar, 10 μm). Data are presented as mean ± SEM from three independent experiments. **(H,I)** Chromosome spreading of TRF1-depleted MII oocytes. Kinetochores and DNA were stained with ACA and DAPI, respectively (scale bar, 20 μm). The incidence of aneuploidy was quantified. Data are presented as mean ± SEM. *p < 0.05, **p < 0.01, ***p < 0.001.

To further investigate the effect of TRF1 depletion on meiotic maturation, we examined spindle and chromosome organization. While typical barrel-shaped spindles with well-aligned chromosomes on the equatorial plate were observed predominantly in control oocytes, TRF1 depletion frequently resulted in spindle assembly defects and chromosome misalignment (spindle abnormality: 12.73 ± 3.11, *n* = 44 vs. 27.78 ± 2.26, *n* = 47, *p* < 0.05; misaligned chromosome: 18.75 ± 3.61, *n* = 44 vs. 55.27 ± 6.79, *n* = 47, *p* < 0.001) (Figures 2A–C). Moreover, the metaphase chromosome plate became considerably wider after TRF1 depletion (11.71 ± 0.35, *n* = 44 vs. 14.84 ± 0.59, *n* = 47, *p* < 0.0001) (Figures 2A,D). This remarkable increase in spindle defects and misaligned chromosomes led us to investigate kMT attachment. In turn, oocytes were cold-treated to depolymerize unstable microtubules not attached to kinetochores. While kinetochores remained fully attached by spindle microtubules in control oocytes, TRF1-depleted oocytes displayed a higher proportion of unattached kinetochores (unattached kinetochore: 4.64 ± 0.91, *n* = 40 vs. 27.74 ± 6.63, *n* = 40, *p* < 0.001; oocyte with abnormal kMT attachment: 28.57 ± 8.24, *n* = 40 vs. 76.19 ± 9.52, *n* = 40, *p* < 0.05) (Figures 2E–G). Because chromosome misalignment and lack of kMT attachments are associated highly with aneuploidy, we performed karyotypic analysis of MII oocytes by chromosome spreading. While a large majority of control oocytes was euploid and had the correct number of univalents, the incidence of aneuploidy significantly increased in TRF1-depleted oocytes (7.45 ± 0.4, *n* = 50 vs. 24.93 ± 4.29, *n* = 50, *p* < 0.05) (Figures 2H,I). Therefore, our results suggest that TRF1 is required to ensure proper chromosome segregation by regulating kMT attachment and the SAC during meiotic maturation in oocytes.

### TRF1 Depletion Impairs Inner Centromere and Kinetochore Assembly but Not CENP-A Assembly at Centromeres

**FIGURE 3:**
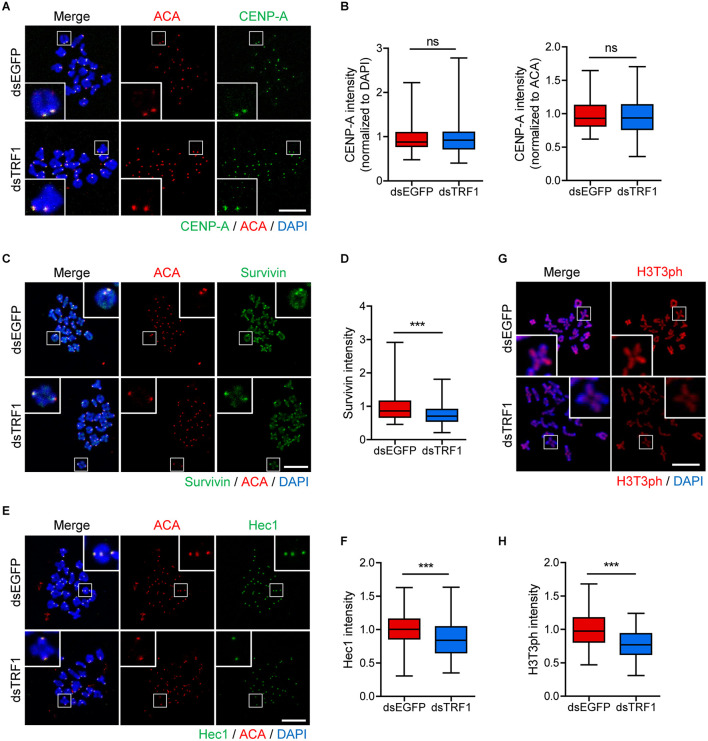
TRF1 is required to maintain inner centromere and kinetochore integrity. Chromosome spreads were prepared from oocytes at the MI stage and were stained with anti-CENP-A, anti-Survivin, anti-Hec1, and anti-H3T3ph antibodies. Kinetochores and DNA were labeled with anti-centromere antibody (ACA) and DAPI, respectively (scale bar, 20 μm). **(A–H)** The intensities of CENP-A (normalized to DAPI or ACA), Survivin, and Hec1 were quantified, also shown in representative images. ns; not significant, ***p < 0.001.

Because telomeres are located in close proximity to centromeres in mouse telocentric chromosomes, we investigated the effects of TRF1 depletion on centromere and kinetochore architectures. Therefore, we immunostained oocytes for CENP-A, Survivin, and Hec1 as centromere, inner centromere, and kinetochore markers, respectively. Interestingly, CENP-A level was indistinguishable between control and TRF1-depleted oocytes (CENP-A intensity normalized to DAPI: 1.0 ± 0.02, *n* = 213 vs. 0.95 ± 0.02, *n* = 172, *p* > 0.05; CENP-A intensity normalized to ACA: 0.98 ± 0.01, *n* = 213 vs. 0.96 ± 0.01, *n* = 172, *p* > 0.05) (Figures 3A,B), suggesting that CENP-A assembly at centromeres is not affected by TRF1 depletion. In stark contrast to CENP-A, Survivin localized at the inter-chromatid axis of bivalents, with strong enrichment at centromeres, significantly decreased after TRF1 depletion (1.0 ± 0.02, *n* = 313 vs. 0.75 ± 0.01, *n* = 238, *p* < 0.0001) (Figures 3C,D). In addition, outer kinetochore protein Ndc80/Hec1 significantly decreased after TRF1 depletion (1.0 ± 0.01, *n* = 334 vs. 0.88 ± 0.02, *n* = 233, *p* < 0.0001) (Figures 3E,F). Because inner centromere localization of Survivin is largely dependent on H3T3ph (Kelly et al., 2010), we asked whether the decrease in Survivin on chromosomes after TRF1 depletion was associated with H3T3ph level. Notably, TRF1 depletion decreased the overall intensity of H3T3ph on chromosomes (1.0 ± 0.02, *n* = 104 vs. 0.77 ± 0.02, *n* = 117, *p* < 0.0001) (Figures 3G,H). Therefore, our results suggest that TRF1 regulates the H3T3ph-dependent inner centromeric localization of Survivin as well as kinetochore assembly but not CENP-A centromere assembly.

### TRF1 Depletion Decreases SMC3 and SMC4 Levels on Chromosomes

**FIGURE 4:**
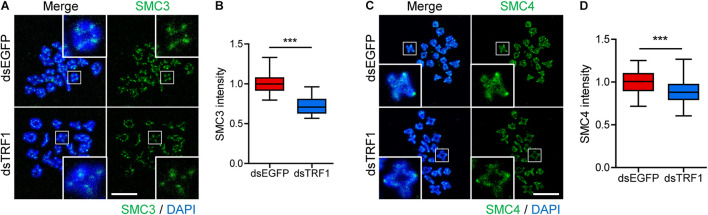
TRF1 is associated with chromosome condensation and cohesion. **(A–D)** Chromosome spreads were prepared from oocytes at the MI stage and were stained with anti-SMC3 and anti-SMC4 antibodies. DNA was counterstained with DAPI. The normalized intensity of SMC3 and SMC4 was quantified and shown with the representative images (scale bar, 20 μm). ***p < 0.001.

Loss of inner centromere-localized Aurora B/C kinases weakens centromeric cohesion, increasing premature chromosome segregation (Ruchaud et al., 2007). Because TRF1 depletion impairs chromosome segregation and Survivin recruitment, we next investigated sister chromatid cohesion in TRF1-depleted oocytes. As reported (Revenkova et al., 2010), the cohesin component SMC3 is located primarily on the inter-chromatid axis of bivalents in control oocytes. However, TRF1 depletion decreased the overall intensity of SMC3 on the chromosomes but did not change the inter-chromatid localization of SMC3 (1.0 ± 0.01, *n* = 69 vs. 0.72 ± 0.03, *n* = 59, *p* < 0.0001) (Figures 4A,B). In addition to cohesin, condensin plays primary roles in chromosome assembly and segregation. Therefore, we examined the condensin subunit SMC4 after TRF1 depletion. Interestingly, TRF1 depletion also decreased the level of SMC4 (1.0 ± 0.01, *n* = 66 vs. 0.88 ± 0.02, *n* = 59, *p* < 0.0001) (Figures 4C,D), suggesting that TRF1 not only regulates inner centromere and kinetochore assembly, but also influences chromosome compaction. Taken together, our results suggest that TRF1 is required to maintain chromosome structural integrity during meiotic maturation in mouse oocytes.

### Inhibition of Inner Centromere or Kinetochore Function Decreases TRF1 Localization to Proximal Chromosome Ends

**FIGURE 5:**
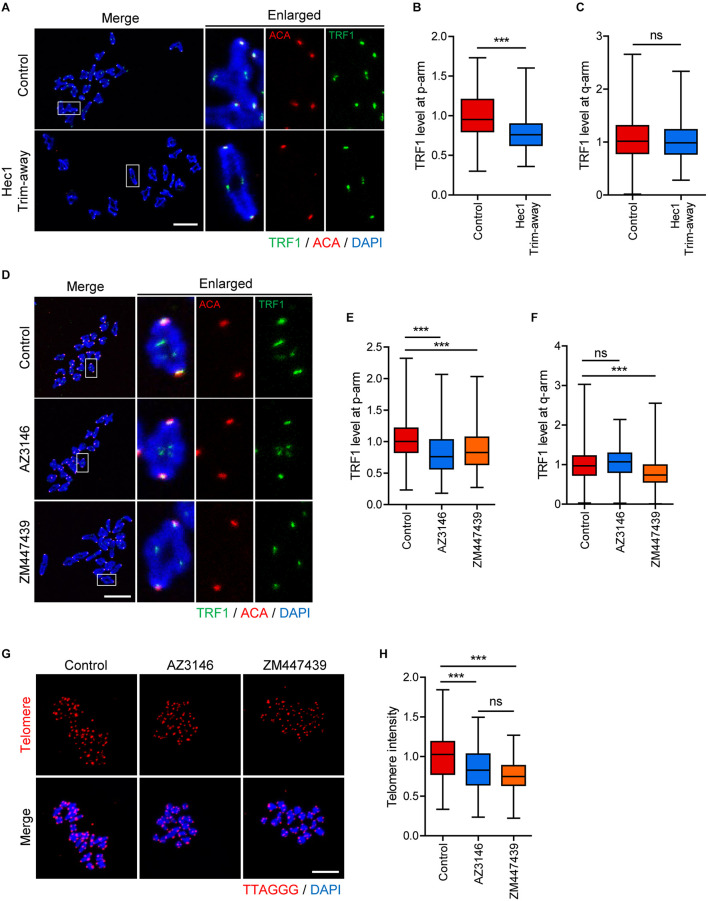
Inhibition of inner centromere or kinetochore function decreases TRF1 level at telomeres. **(A–C)** Ndc80/Hec1 chromosome spreads were prepared from MI oocytes depleted Hec1 using the Trim-away method, and chromosome spreads were stained with anti-TRF1 antibody. Kinetochores and DNA were labeled with anti-centromere antibody (ACA) and DAPI, respectively (scale bar, 20 μm). **(A)** Representative images from three independent experiments are shown. **(B,C)** The normalized intensity of TRF1 at p-arms and q-arms was quantified. **(D–F)** Chromosome spreads were prepared from MI oocytes after treatment with DMSO (Control), AZ3146, or ZM447439 and stained with anti-TRF1 antibody. Kinetochores and DNA were labeled with anti-centromere antibody (ACA) and DAPI, respectively (scale bar, 20 μm). **(D)** Representative images from three independent experiments are shown. **(E,F)** The normalized intensity of TRF1 at p-arms and q-arms was quantified. **(G,H)** After treating with DMSO (Control), AZ3146, or ZM447439, oocytes were cultured for 12 h and subjected to telomere Q-FISH. Relative telomere FISH intensity was quantified and shown with representative images (scale bar, 20 μm). ns; not significant, ***p < 0.001.

Although the phenotype analysis of TRF1 depletion indicated that TRF1 was required for kinetochore assembly, it is unknown whether kinetochore function is required to maintain telomere integrity. Therefore, we sought to disrupt kinetochore function by depleting Ndc80/Hec1. Because Ndc80/Hec1 depletion severely impairs GVBD (Gui and Homer, 2013), we employed Trim-away, a newly developed method for depleting rapidly and specifically proteins in oocytes, to remove Ndc80/Hec1 after GVBD (Clift et al., 2017). Immunostaining analysis showed that Ndc80/Hec1 levels were significantly reduced following the Trim-away method (1.0 ± 0.03, *n* = 35 vs. 0.34 ± 0.06, *n* = 40, *p* < 0.0001) (Supplementary Figure 3). Interestingly, TRF1 level at the proximal end (p-arm) significantly decreased after Ndc80/Hec1 depletion, but that at the distal end (q-arm) was not affected by Ndc80/Hec1 depletion (TRF1 level at p-arm: 1.0 ± 0.02, *n* = 104 vs. 0.77 ± 0.01, *n* = 218, *p* < 0.0001; TRF1 level a q-arm: 1.08 ± 0.03, *n* = 208 vs. 1.01 ± 0.02, *n* = 214, *p* > 0.05) (Figures 5A–C). This result suggests that kinetochore function affects proximally adjacent telomeres. Consistent with this, inhibition of kinetochore kinase Mps1 using AZ3146 also decreased TRF1 at p-arms but not at q-arms (TRF1 level at p-arm, control: 1.01 ± 0.01, *n* = 279; AZ3146: 0.82 ± 0.02, *n* = 246, *p* < 0.0001; ZM447439: 0.86 ± 0.02, *n* = 232, *p* < 0.0001; TRF1 level a q-arm, control: 1.00 ± 0.02, *n* = 377; AZ3146: 1.06 ± 0.02, *n* = 380, *p* > 0.05; ZM447439: 0.79 ± 0.02, *n* = 401, *p* < 0.0001) (Figures 5D–F). Moreover, Mps1 inhibition led to a partial decrease in telomere length (Figures 5G,H).

Because TRF1 depletion impaired inner centromere localization of Survivin, we asked whether inner centromere function was involved in telomere integrity and length. Therefore, oocytes were treated with Aurora kinase inhibitor ZM447439, and TRF1 level and telomere length were determined. Notably, TRF1 level at both p-arm and q-arm telomeres significantly decreased following ZM447439 treatment (Figures 5D–F). Consistent with the decrease in TRF1 level, ZM447439 treatment resulted in a significant decrease in telomere length (control: 1.00 ± 0.02, *n* = 102; AZ3146: 0.83 ± 0.02, *n* = 107, *p* < 0.0001; ZM447439: 0.76 ± 0.01, *n* = 101, *p* < 0.0001) (Figures 5G,H), suggesting that inner centromere function is required to maintain telomere integrity. Taken together, our results suggest that telomeres, kinetochores, and inner centromeres mutually regulate each other in mouse oocyte telocentric chromosomes.

## Discussion

Centromeres and telomeres, which are composed of highly repetitive heterochromatin, are two specialized chromosomal structures that are essential for chromosome stability and segregation. The centromeres interact with spindle microtubules to ensure the proper segregation of sister chromatids and homologous chromosomes during mitosis and meiosis. On the other hand, telomeres are the end regions of chromosomes that help maintain genomic stability by protecting chromosomal ends from erosion and end-to-end fusion. In telocentric mouse chromosomes, centromeres and telomeres are located in close proximity, raising the possibility of functional crosstalk between the two structures. In this study, we found that maintaining telomere integrity was required to preserve inner centromere and kinetochore architectures, and vice versa.

We found that TRF1 depletion caused precocious PBE, impairing the recruitment of SAC proteins at the kinetochores. Consistent with our results, TRF1 has been shown to colocalize with SAC proteins BubR1, Mad1, and Mad2 at mouse telomeres (Prime and Markie, 2005; Munoz et al., 2009). Moreover, TRF1 depletion led to a decrease in Survivin localization to the inner centromere. Given that Survivin forms complexes with Aurora B/C kinases, TRF1 depletion might abolish the inner centromeric recruitment of Aurora B/C kinases. Indeed, TRF1 is required for the centromeric function of Aurora B kinase during mitosis in HeLa cells (Ohishi et al., 2014). In this regard, we speculate that the phenotypes derived from TRF1 depletion are highly associated with impaired Aurora B/C kinase function at the inner centromere. In addition to SAC proteins and Survivin, TRF1 depletion impaired Ndc80/Hec1 recruitment at the outer kinetochore. Conversely, Ndc80/Hec1 depletion decreased TRF1 level at telomeres, suggesting mutual interaction between TRF1 and Ndc80/Hec1. Although TRF1 could directly interact with several proteins, including SAC (Prime and Markie, 2005; Munoz et al., 2009), TRF1 has not been reported to be associated with Survivin in complex with Aurora B/C kinases and the outer kinetochore KMN network. Therefore, considering that TRF1 is crucial for the maintenance of telomere integrity, it is possible that Survivin and Ndc80/Hec1 depletion is an indirect consequence of reduced telomere integrity induced by TRF1 depletion rather than by direct interaction between TRF1 and Ndc80/Hec1 or Survivin. In contrast to Survivin and Ndc80/Hec1 levels, CENP-A level did not change after TRF1 depletion. This is consistent with a previous report demonstrating the remarkable stability of CENP-A in oocytes that persisted long after its deposition at centromeres (Smoak et al., 2016). Therefore, we suggest that TRF1-mediated maintenance of telomere integrity is essential for inner centromere and kinetochore functions, and vice versa. We also found that TRF1 depletion decreased cohesin and condensin components SMC3 and SMC4, respectively, implying that telomere integrity is associated with chromosome cohesion and condensation. This is supported by the observation that condensin is enriched in the vicinity of telomeres (Viera et al., 2007; Kim et al., 2013). Moreover, the condensin component NCAPH2 colocalizes and interacts with TRF1 at the telomere (Wallace et al., 2019). In addition to condensin, cohesin SMC1β and SMC3 have been shown to localize at telomeres and prevent telomere shortening (Adelfalk et al., 2009). Thus, it is likely that telomere integrity is required not only to protect chromosome ends from fusion and erosion, but also to preserve chromosome integrity, such as proper cohesion and condensation.

It is well established that aneuploidy is the leading cause of poor reproductive outcomes, including implantation failure and miscarriage (Zona Pellucida Glycoproteins and Immunocontraception., 1996). Moreover, numerous studies have reported that aneuploidy in oocytes increases with maternal age (Merriman et al., 2012; Cheng et al., 2016; Camlin et al., 2017). Although many factors have been suggested as possible causes of aneuploidy, the deterioration of chromosome cohesion with increasing maternal age is a leading cause of age-associated aneuploidy (Lister et al., 2010; Chiang et al., 2012; Jessberger, 2012). Given that telomere shortening is one of the prominent hallmarks of aging (Hanna et al., 2009), it is possible to speculate that the age-related increase in aneuploidy is caused by a decrease in telomere integrity with age. Indeed, we found that TRF1 depletion significantly decreased cohesin SMC3 level. However, TRF1 decreased either by depleting Ndc80/Hec1 or by inhibiting Aurora B/C kinases. Therefore, we could not exclude the possibility that the age-associated cohesion loss is the initial event that triggers a reduction in the integrity of centromeres as well as kinetochores, which in turn would impair the recruitment of SAC and Aurora B/C kinases at the outer kinetochores and inner centromeres, respectively, and subsequently cause telomere shortening. Consistent with this, it has been reported that aging causes a significant kinetochore-associated loss in SAC proteins and phosphorylated Aurora C in mouse oocytes (Yun et al., 2014). Taken together, these findings indicate reciprocal regulation between components that regulate chromosome organization, which deteriorates with maternal aging.

## Conclusion

Our data demonstrate that TRF1-mediated maintenance of telomere integrity is required to preserve centromere and kinetochore integrity as well as to maintain chromosome cohesion and condensation. Conversely, centromere and kinetochore functions were demonstrated to be necessary for the maintenance of telomere integrity, suggesting mutual regulation between chromosomal structures in telocentric mouse chromosomes.

## Data Availability Statement

The original contributions presented in the study are included in the article/Supplementary Material, further inquiries can be directed to the corresponding author.

## Ethics Statement

The animal study was reviewed and approved by Institutional Animal Care and Use Committees of Sungkyunkwan University (approval ID: SKKUIACUC2020-05-01-1).

## Author Contributions

H-JJ and JO conceived and designed the experiments. H-JJ performed all experimentation and data analysis. JO supervised the study and wrote the manuscript. Both authors contributed to the article and approved the submitted version.

## Conflict of Interest

The authors declare that the research was conducted in the absence of any commercial or financial relationships that could be construed as a potential conflict of interest.

## Publisher’s Note

All claims expressed in this article are solely those of the authors and do not necessarily represent those of their affiliated organizations, or those of the publisher, the editors and the reviewers. Any product that may be evaluated in this article, or claim that may be made by its manufacturer, is not guaranteed or endorsed by the publisher.
