# Supplementary material for: TRF1 Depletion Reveals Mutual Regulation Between Telomeres, Kinetochores, and Inner Centromeres in Mouse Oocytes
Source: Front Cell Dev Biol. 2021 Sep 17;9:749116. doi: 10.3389/fcell.2021.749116 (PMC8486315; doi:10.3389/fcell.2021.749116)
Supplement: Supplementary file 1 [file Data_Sheet_1.PDF]

Fig. S1

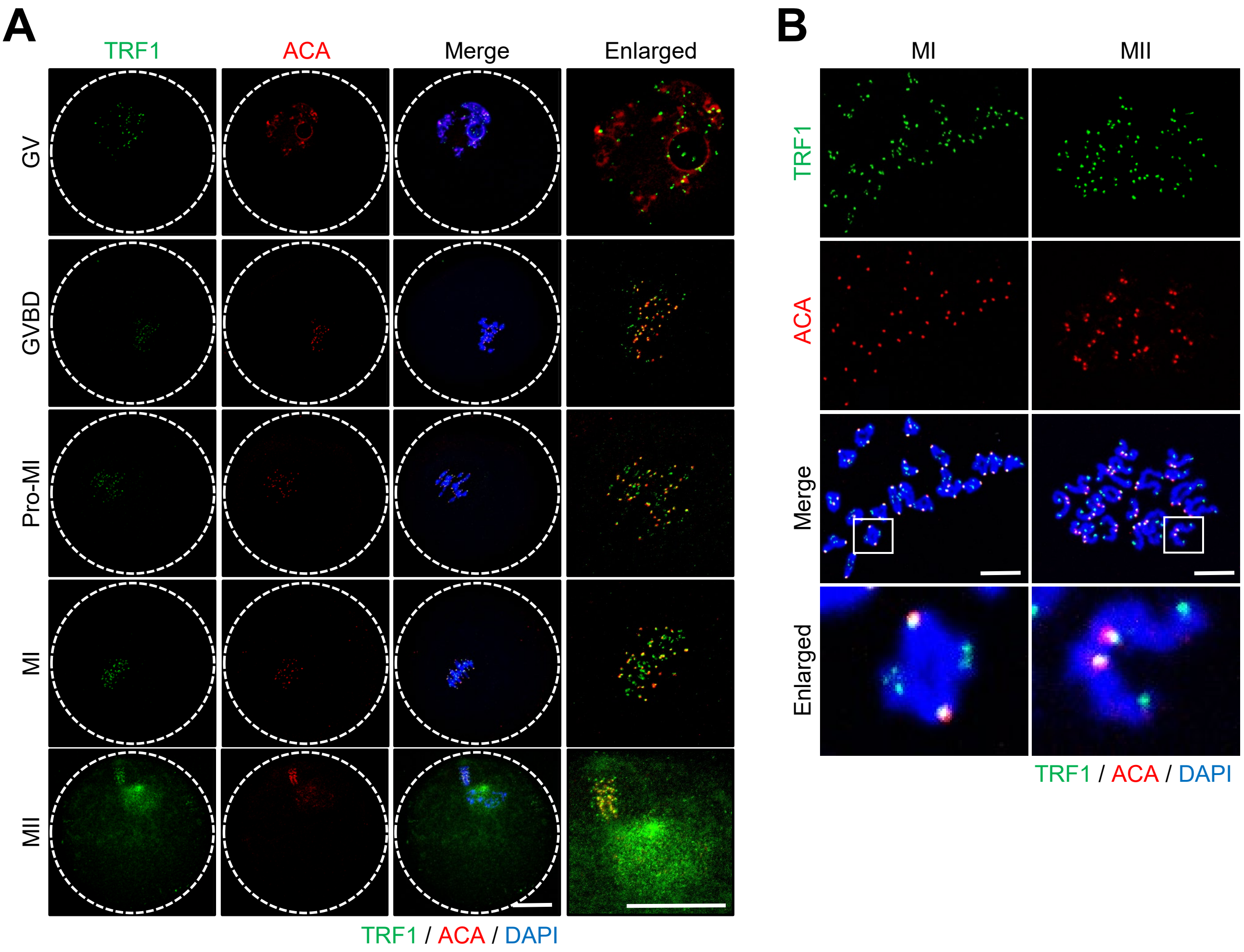

Fig. S1. Localization of TRF1 in mouse oocytes during meiotic maturation. (A) Oocytes at GV, GVBD, Pro-MI, MI, and MII stage were fixed and stained with anti-TRF1 antibodies. Kinetochores were labeled with anti-centromere antibodies (ACA). DNA was counterstained with DAPI. Scale bar, 20 μm. (B) Chromosome spreads were prepared from MI and MII oocytes and stained with anti-TRF1 antibodies. Kinetochores were labeled with anti-centromere antibodies (ACA). DNA was counterstained with DAPI. Scale bar, 20 μm. GV, germinal vesicle; GVBD, germinal vesicle breakdown; Pro-MI, prometaphase I; MI, metaphase I; MII, metaphase II.

Fig. S2

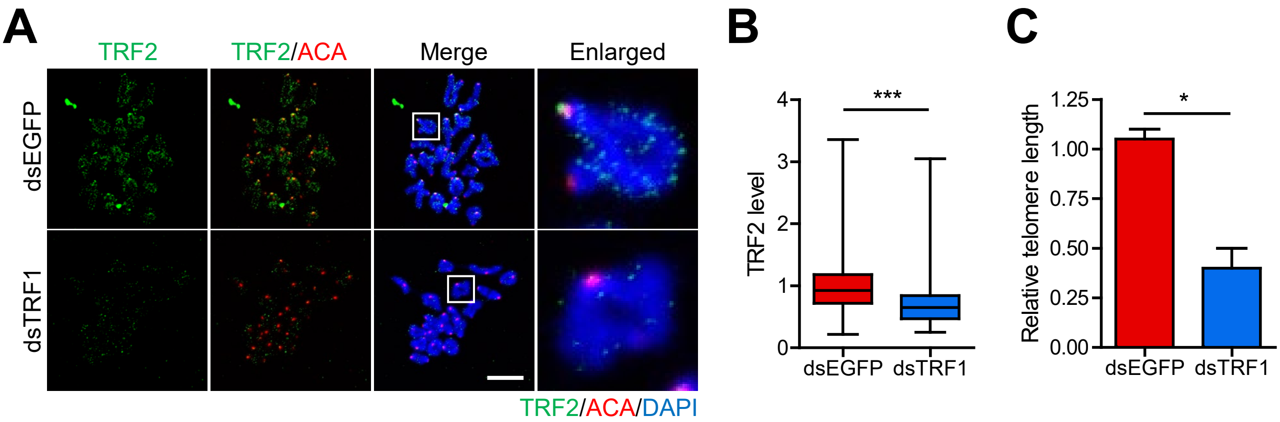

Fig. S2. TRF1 depletion causes a partial loss of TRF2 and telomere shortening in mouse oocytes. Oocytes were injected with dsEGFP or dsTRF1 and cultured for 24 h in the IBMX-containing medium. After release from IBMX-mediated GV arrest, oocytes were cultured to MI stage, then chromosomes were spread and stained with anti-TRF2 antibody. Kinetochores were labeled with anti-centromere antibodies (ACA). DNA was counterstained with DAPI. Scale bar, 20 μm. (B) TRF2 intensity was quantified and normalized to ACA level. (C) Relative telomere length was measured by telomeric Q-PCR. The data are expressed as mean ± SEM from three independent experiments. *$p<0.05$; ***$p<0.001$.

Fig. S3

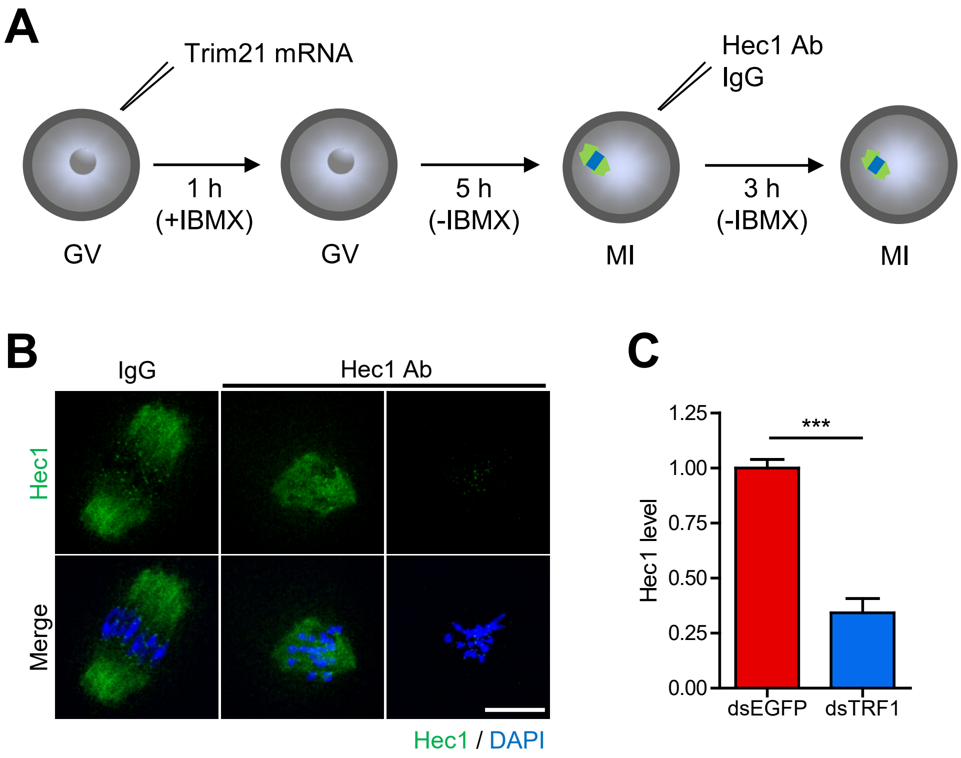

Fig. S3. Hec1 depletion using Trim-away method. (A) Schematic of Hec1 Trim-away method. Trim21 mRNA was microinjected into GV oocytes. After 1 h culture with IBMX for mRNA expression, GV oocytes were released from IBMX-mediated arrest. After 5 h culture in IBMX-free medium, MI oocytes were injected with either Hec1 antibody (Ab) or IgG and cultured for 3 h to allow Hec1 degradation. (B) Immunostaining of Hec1 in Hec1 Trim-away oocytes. DNA was counterstained with DAPI. Scale bar, 20 μm. (C) Hec1 intensity was quantified and normalized to mean intensity of DAPI signal. ***p<0.001.
